# Aggregate Removal Nanofiltration of Human Serum Albumin Solution Using Nanocellulose-Based Filter Paper

**DOI:** 10.3390/biomedicines8070209

**Published:** 2020-07-13

**Authors:** Lulu Wu, Athanasios Mantas, Simon Gustafsson, Levon Manukyan, Albert Mihranyan

**Affiliations:** Nanotechnology and Functional Materials, Department of Materials Science and Engineering, Box 35, Uppsala University, 751 03 Uppsala, Sweden; lulu.wu@angstrom.uu.se (L.W.); athanasios.mantas@angstrom.uu.se (A.M.); simon.j.gustafsson@gmail.com (S.G.); levon.manukyan@angstrom.uu.se (L.M.)

**Keywords:** virus removal filtration, *Cladophora* cellulose, plasma-derived biologics, cell culture, cell therapies

## Abstract

This study is dedicated to the rapid removal of protein aggregates and viruses from plasma-derived human serum albumin (HSA) product to reduce the risk of viral contamination and increase biosafety. A two-step filtration approach was implemented to first remove HSA aggregates and then achieve high model virus clearance using a nanocellulose-based filter paper of different thicknesses, i.e., 11 μm (prefilter) and 22 μm (virus filter) at pH 7.4 and room temperature. The pore size distribution of these filters was characterized by nitrogen gas sorption analysis. Dynamic light scattering (DLS) and size-exclusion high performance liquid chromatography (SE-HPLC) were performed to analyze the presence of HSA aggregates in process intermediates. The virus filter showed high clearance of a small-size model virus, i.e., log_10_ reduction value (LRV) > 5, when operated at 3 and 5 bar, but a distinct decrease in LRV was detected at 1 bar, i.e., LRV 2.65–3.75. The throughput of HSA was also dependent on applied transmembrane pressure as was seen by Vmax values of 110 ± 2.5 L m^−2^ and 63.6 ± 5.8 L m^−2^ at 3 bar and 5 bar, respectively. Protein loss was low, i.e., recovery > 90%. A distribution of pore sizes between 40 nm and 60 nm, which was present in the prefilter and absent in the virus filter, played a crucial part in removing the HSA aggregates and minimizing the risk of virus filter fouling. The presented results enable the application of virus removal nanofiltration of HSA in bioprocessing as an alternative to virus inactivation methods based, e.g., on heat treatment.

## 1. Introduction

Human serum albumin (HSA) is one of the most important products derived from human plasma. It has a multifunctional role as an osmotic pressure regulator, transport shuttle and redox modulator [1]. HSA is used as an important component for producing drug-protein conjugates, e.g., for cancer treatment, due to its long blood circulation half-life [2] and accumulation in tumors [3]. HSA is also a critical supplement for cell culture media intended for cell therapies [4] and a widely used cryoprotectant for cells [5].

In recent years, there has been a sustained interest in developing recombinant HSA, which is commercially available in limited quantities for applications as (i) a cryoprotectant for other recombinant biologicals; (ii) a component in serum-free cell culture media for cell therapies; and (iii) a specialty component in diagnostic imaging agents [6]. The main challenges for recombinant HSA so far include the cost-efficiency of large-scale manufacturing and product purity [7,8].

Because most available HSA is still derived from human plasma, this infers the risk of virus contamination. Donor screening is the first step to mitigate the risk of viral contamination. The next step of assuring HSA’s biosafety is pasteurization, which is typically performed at 60 °C for 10 h in the presence of stabilizers, such as N-acetyl tryptophan and/or caprylate (octanoate) [9]. Another stabilizer, i.e., N-acetyl-L-methionine, was reported as an alternative to N-acetyl-tryptophan as an HSA stabilizer [10,11]. The time of pasteurization is critical for efficient inactivation of virus particles, e.g., 4 h pasteurization may not be sufficient to inactivate hepatitis B viruses as opposed to a 10 h treatment [12]. While the method has been widely accepted in manufacturing of HSA in industry, the emergence of new viruses, e.g., Zika virus and Chikungunya virus, stipulates constant revalidation of the process [13,14,15,16,17]. Pasteurization of HSA (60 °C, 10 h) was shown to reduce the infectivity of most known human viruses including human parvovirus B19, hepatitis A virus, human immunodeficiency virus and West Nile virus [18,19,20,21], but it may be ineffective against animal parvoviruses, e.g., canine parvovirus (CPV) and minute virus of mice (MVM) [21]. The presence of N-acetyl-tryptophan or caprylate in HSA has little effect on inactivation kinetics during pasteurization, unlike other blood-derived products which may be stabilized by different excipients such as sucrose and CaCl_2_. [21]. However, the presence of high quantities of HSA stabilizers (e.g., typical stabilizer: Albumin molar ratio > 5:1) negatively affects the binding and transport properties of HSA, especially with respect to lipophilic molecules [22,23]. Further, pasteurized HSA does not have the redox properties of native HSA and contains higher quantities of cysteinylated, i.e., Cys34-bound, albumin, especially S-nitrosoalbumin [24]. Moreover, excessive amounts of stabilizers can lead to undesired biological side effects. For instance, both caprylate and N-acetyl-tryptophan have been identified as vasodilators and may contribute to reduced renal perfusion [25]. Caprylate used as a stabilizer for HSA supplement was found detrimental for mesenchymal stem cell growth and differentiation [26]. In all, it would be advantageous to develop virus clearance processes that would eliminate the need for pasteurization and thereby remove the need for stabilizers.

Virus removal filtration is a well-established method of filtering biologicals thanks to its inertness and proven removal performance. However, virus removal filtration of HSA is rarely used due to cost-efficiency issues. Another technical challenge with HSA filtration is its tendency to form aggregates during pasteurization, which leads to rapid clogging of filters. It has been shown that when fatty acids are bound to HSA, its structure does not unfold upon heating even upon extended heating [27]. Since stabilizers dramatically increase structural stability of HSA upon heating, aggregate formation upon heating proceeds due to alternative pathways. In particular, it is believed to be associated with carry-over impurities, which are present in plasma-derived HSA, e.g., haptoglobin, transferrin, Gc-globulin and β2-glycoprotein, and which unfold upon heating and thereby promote aggregate formation [28,29]. In this respect, it should be noted that even though clinical-grade HSA is considered to be pure (≥95–96%), there are traces of as many as 141 different proteins other than HSA [30]. Chemical and immunochemical analysis of HSA aggregates from commercial vendors shows that these aggregates contain only 30–50% HSA, whereas the rest is made from denatured thermolabile proteins, i.e., mainly haptoglobin [28,31,32]. To confirm the deleterious effect of impurities, it was reported that affinity chromatographic (concanavalin A) removal of haptoglobin and hemopexin greatly reduces the aggregate formation tendency of pasteurized HSA [29]. Aggregate formation mainly proceeds through disulfide bonds between albumin and small amounts of denatured impurity globulins during the pasteurization step. It has earlier been reported that capping the free thiol group in bovine serum albumin (BSA) molecules with cysteine induces a remarkable decrease in the amount of the BSA aggregates during ultrafiltration [33]. Overall, aggregate products present in HSA negatively affect filterability of albumin.

In recent years a new type of virus removal filter paper has been developed at Uppsala University, which combines desirable pathogen removal properties with high cost-efficiency. Using naturally-sourced cellulose nanofibers, a nonwoven filter paper was produced via conventional hot-pressing of wet pulp [34]. The resultant paper featured a pore size mode of 19 nm, which enabled removal of surrogate nanoparticles such as fluorescently-labeled latex nanobeads of varying size, and a real virus, i.e., swine influenza virus A [34]. The virus removal capability was further confirmed using large-size model viruses such as retroviruses (100 nm; enveloped), i.e., xenotropic murine leukemia virus (xMuLV), with excellent clearance, i.e., log_10_ reduction value (LRV) ≥ 5.25 [35]. The potential of using nanocellulose-based filter paper for viral clearance was ultimately confirmed for the worst-case small-size model viruses, i.e., parvoviruses (20 nm, nonenveloped) [36].

The produced filter paper consists of numerous stacked nanosheets formed via self-assembly of cellulose nanofibers during drainage, wet-cake formation and then hot-press drying, giving rise to -called mille-feuille structure [36]. By controlling the evaporation rate of moisture, the pore size mode can be varied between 10 and 25 nm [37]. The latter means that it is possible to remove particles of a certain size in solution by controlling the pore size distribution of the nanocellulose-based filter paper, something that will be explored in this manuscript.

Possible applications of mille-feuille filter paper in downstream and upstream bioprocessing purification have been studied. For upstream bioprocessing, filtration of virus spiked basal media, e.g., Dulbecco’s modified Eagle’s medium (DMEM), LRV ≥ 5 was shown for small-size ΦX174 phages [38]. The filters were also found useful for filtering chemically-defined Chinese hamster ovary (CHO) cells medium supplemented with insulin-transferrin-selenium (ITS) and containing Pluronic F-68 [39]. In addition to high virus retention capacity and good flow rates, the results also showed no impact on cell viability, morphology and confluence [39]. When applied in downstream bioprocessing, the filter exhibited 5–6 LRV of ΦX174 (28 nm) or MS2 (27 nm) phages during the filtration of spiked human plasma-derived intravenous immunoglobulin (IVIG) at 3 bar [40]. Recently, the filtration of plasma-derived human coagulation factor IX-rich prothrombin complex with mille-feuille filter paper was shown [41].

In this article the virus removal properties for filtration of plasma-derived HSA are explored. We present a two-step sequential filtration of HSA solution through nanocellulose filters of different thicknesses, i.e., first 11 μm (prefilter) and then 22 μm (virus removal filter), which enable size-exclusion based removal of undesirable protein aggregates and achieve high protein throughput as well as high virus clearance. The filters were made of identical material and showed only slightly different pore size distributions.

## 2. Materials and Methods

### 2.1. Materials

Human serum albumin (HSA) (200 mg mL^−1^; ≥96% albumin) was purchased from a local apothecary store and contained the following excipients: sodium chloride, N-acetyl-tryptophan and caprylic acid. Phosphate buffer saline (PBS) was purchased from Sigma-Aldrich (Saint Louis, MO, USA). Total protein biuret reagent and sodium chloride were obtained from Sigma-Aldrich (Saint Louis, MO, USA). *Escherichia coli* bacteriophage ΦX174 (ATCC^®^ 13706) and *Escherichia coli* (Migula) Castellani and Chalmers (*E. coli*) (ATCC^®^ 13706-B1) were obtained from American Type Culture Collection (ATCC, Manassas, VA, USA). Yeast extract, tryptone and agar were obtained from Becton, Dickinson and Company (BD, Franklin Lakes, NJ, USA). Cellulose from *Cladophora* sp. algae, collected from Qingdao, China, was provided by Dr. Jun Liu, Jiangsu University.

### 2.2. Methods

#### Filter Paper Preparation

*Cladophora* cellulose raw material (0.2% wt.) was predispersed in deionized water using an IKA T25 high-shear mixer (Staufen, Germany) prior to high pressure-homogenization. The final nanocellulose dispersion was prepared by passing the mixed cellulose suspension through a high-pressure LM20 microfluidizer (Microfluidics, MA, USA). The dispersion was passed 3 times through a 200-µm grid chamber and 1 time through a 100-µm grid chamber under a pressure of 1800 bar.

The nanocellulose-based filters were prepared as previously described [39]. Briefly, the diluted dispersion was drained through a membrane (0.65 µm hydrophilic polyvinylidene difluoride

PVDF; Merck Millipore, MA, USA) using a vacuum filtration setup (Advantec, Dublin, CA, USA) until a cellulose cake was formed. The wet cake was then dried in a Carver 4122CE press (Carver, IN, USA). For the preparation of 11 μm thick prefilters, the nanocellulose wet-cake was dried at 140 °C using a hot-press for 40 min. For the preparation of the 22 μm thick virus removal filter, the nanocellulose wet cake was dried at 80 °C using a hot-press for 24 h. The dry filters were removed, cut into 47 mm diameter disc, and stored at ambient conditions until further use.

### 2.3. Filtration

#### 2.3.1. Filtration Setup

An Advantec KST-47 (Advantec, Dublin, CA, USA) filter holder was used. A general-purpose filter paper disc 47 mm in diameter (Munktell, Eskilstuna, Sweden) was placed beneath the nanocellulose filter as a support. The rate of flow was monitored gravimetrically by collecting the outflowing liquid on an MS1602TS analytic balance (Mettler Toledo, Columbus, OH, USA) connected to LabX software (Version 2.5, Mettler Toledo, Switzerland).

#### 2.3.2. Prefiltration

Eleven µm filters were used for prefiltration. Feed solution was 10 mg mL^−1^ HSA diluted in PBS (10 mM) adjusted to pH 7.4. The filters were wetted with PBS prior to prefiltration. Prefiltrations were carried out at 1 bar. Due to rapid clogging, for each prefiltration around 25 mL was passed through each filter, corresponding to a 14.4 L m^−2^ load volume. The permeate fractions were collected, mixed together and stored at 4 °C before use.

#### 2.3.3. Virus Removal Filtration

ΦX174 bacteriophage (28 nm) was used as a model small-size virus. Twenty-two µm filters were used for virus removal studies. A stock solution of ΦX174 was spiked at 0.1% into prefiltered 10 mg mL^−1^ HSA in PBS adjusted to pH 7.4. Virus stability was controlled by a hold sample taken from the prefiltered spiked feed solution. The filters were wetted with PBS prior to filtration. Filtrations were carried out at 3 different overhead pressures, i.e., 1, 3, and 5 bar. For each pressure, around 50 mL of the entire loaded feed solution was passed through the filters, corresponding to a 28.8 L m^−2^ load volume. The permeate was collected in two equal fractions. The average flux during filtration was recorded as described above. Permeate samples and hold samples were collected and stored at 4 °C before plaque forming units (PFU) assay. The virus removal efficiency was expressed in log_10_ reduction values (LRVs) as described below.

#### 2.3.4. Vmax Analysis

To quantify the throughput of down-scale virus filtering, Vmax analysis was performed on the resulting flux curve. An intermediate fouling model was selected according to a procedure described in Badmington et al. [42]. Vmax was calculated from the slope of the linear fit associated with each flux data curve where take time (h) was set as the *x*-axis and the reciprocal of the relative flux (L m^−2^ h^−1^) as the *y*-axis for linear fitting. When the resulting slope is positive, the reciprocal of the slope is Vmax (L m^−2^) [42].

#### 2.3.5. Scanning Electron Microscopy

To obtain a neat cross-section, the filter was first immersed in liquid nitrogen then broken with tweezers. After being sputtered with Au/Pd prior (Polaron, Ashford, UK), the samples were observed by a high resolution FEG Zeiss 1550 SEM (ZEISS, Jena, Germany) system. The selected acceleration voltage was set between 1.5 kV and 3 kV and In Lens detector was used for imaging.

#### 2.3.6. Nitrogen Gas Sorption

Prior to analysis, the samples were degassed for 8 h in a vacuum at 95 °C. Nitrogen gas sorption was performed using an ASAP 2020 (Micromeritics, GA, USA) instrument. Pore size distribution profiles were obtained using the Barret-Joyner-Halenda (BJH) method [43], based on the desorption branch of the isotherm curve. Data analysis was done using the manufacturer’s software (ASAP, Micromeritics, GA, USA). The performance of the instrument was validated using a porous standard, i.e., Micrometrics^TM^ Silica-Alumina (SSA 210 m^2^ g^−1^; lot number: A-501-49). The deviation between the pore-size mode of the calibration data from the nominal standard values was 0 nm. Measurement on experimental samples was performed in triplicate, and the results were averaged.

#### 2.3.7. Dynamic Light Scattering

Dynamic light scattering (DLS) was used to assess the particle size distribution of 10 mg mL^−1^ HSA in PBS solution (pH 7.4) with a Zetasizer Nano ZS DLS instrument (Malvern, Malvern, UK). The feed solution, prefiltrate and final permeate were used for analysis. The test angle was 173° (backscatter). About 1.2 mL of the test sample was taken each time and equilibrated in a DLS instrument for 10 min at 25 °C before testing. Three parallel samples of each group were used for testing and the results were averaged.

#### 2.3.8. Size Exclusion High Performance Liquid Chromatography (SE-HPLC)

An HPLC-UV system was used for size-exclusion chromatography. A Hitachi 5160 pump (Hitachi Chromaster, Tokyo, Japan) was used with a 5280 autosampler, and a bioZen 1.8 μm SEC-3 analytical column Mw 10–1500 kDa (Phenomenex, Værløse, Denmark). The column temperature was 25 °C, and injection temperature was 20 °C. The mobile phase was 100 mM sodium phosphate pH 6.8. The flow rate was 0.3 mL min^−1^ and run time was 20 min. UV detection at λ = 280 nm was used.

#### 2.3.9. Protein Recovery

Total protein biuret reagent was used to assess protein recovery post filtration of HSA solutions. Six replicates were made for each sample; 50 μL of sample was mixed with 150 μL total protein reagent and the reaction was protected from light for 10 to 30 min then absorbance was measured at 540 nm using a Tecan M200 microplate reader. The recovery rate was calculated using Equation (1) as follows:

(1) $$R=\frac{{\mathrm{Abs}}_{\mathrm{permeate}}}{{\mathrm{Abs}}_{\mathrm{feed}}}\times 100$$

where R is the protein recovery in percent, Abs_permeate_ and Abs_feed_ are the absorbance values at 540 nm for permeate and feed, respectively. It was ascertained that the concentration-absorbance relationship was linear in the studied range of HSA concentration (r^2^ = 0.99968).

#### 2.3.10. Plaque Forming Units (PFU) and log10 Reduction Value (LRV)

The titer of ΦΧ174 bacteriophage was determined by a plaque forming units (PFU) assay. The feed and permeate samples were serially diluted in Luria-Bertani medium (LBM) (1% tryptone, 0.5% yeast extract, and 1% NaCl in deionized water), and 100 μL of diluted bacteriophage was mixed with 200 μL of *E. coli* stock. The resulting suspension was mixed with 1 mL of melted soft agar and poured on the surface of a prepared hard agar plate (55 × 15 mm) and incubated at 37 °C for 5 h. Bacteriophage titer was calculated using Equation (2):

(2) $$\log_{10}\left(\frac{\mathrm{PFU}}{\mathrm{mL}}\right)=\log_{10}\left(\frac{\mathrm{average}\;\mathrm{number}\;\mathrm{of}\;\mathrm{plaques}}{0.1\;\cdot \;\mathrm{dilution}\;\mathrm{factor}}\right)$$

where 0.1 is the volume (mL) of added virus. The feed titer was adjusted to about 10^5^ to 10^6^ bacteriophages mL^−1^. The limit of detection, i.e., ≤0.7 PFU mL^−1^, of the current experimental design refers to ≤5 bacteriophages mL^−1^, corresponding to a single detectable plaque in one of the plates for nondiluted duplicate samples, assuming that at the detection limit each plaque is produced by one bacteriophage. Virus retention was expressed as LRV (log_10_ reduction value):

(3) $$\mathrm{LRV}=\log_{10}\;\left(\frac{{\mathrm{PFU}}_{\mathrm{feed}}}{{\mathrm{PFU}}_{\mathrm{permeate}}}\right)$$

## 3. Results and Discussion

**Figure 1:**
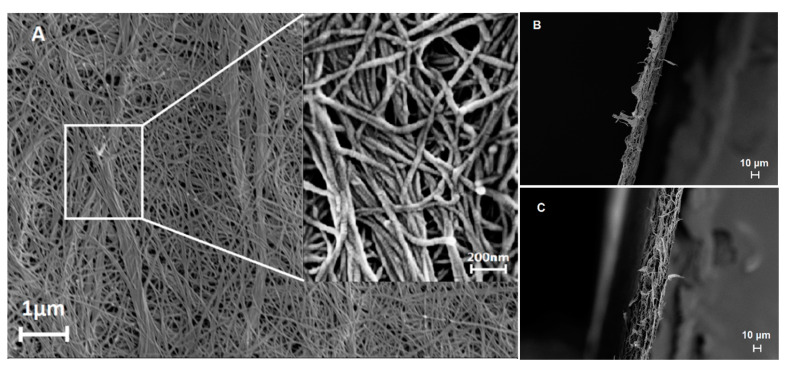
SEM images of prefilter (11 μm) and virus filter (22 μm): (**A**) surface topography (**B**) cross-section of 11 μm prefilter and (**C**) 22 μm virus filter.

Figure 1 shows scanning electron microscope (SEM) images of the studied filters. Figure 1A displays the surface topography of the nanocellulose filter in which the cellulose nanofibers appear stacked and interconnected. It can be seen that many cellulose nanofibers are not completely dispersed and are arranged in a rope-like shape. The insert in Figure 1A shows the magnified structure of the rope-like structures, which appear to be caused by tight winding and tangling of cellulose nanofibers. Compared to the rest of the surface, wherein an open web-like structure is seen, the rope-like structures appear densely packed and do not feature pores. Figure 1B,C shows cross-section images of 11 and 22 μm filters depicting the differences in thickness between the filters. The edges of the cross-section images feature rough fringes, which are most likely caused by the rope-like structures. The rope-like structures overall confer mechanical strength to the filter.

**Figure 2:**
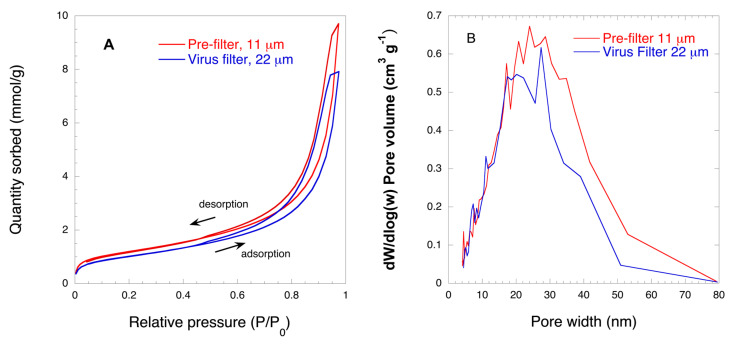
Typical nitrogen gas sorption isotherms (**A**) and average Barret-Joyner-Halenda (BJH) pore size distributions (n = 3) (**B**) for 11 μm prefilter and 22 μm virus filter. Hysteresis loops of the nitrogen sorption isotherms were marked by an upward pointing arrow for adsorption and a downward pointing arrow for desorption.

Figure 2 shows the results of the nitrogen gas sorption analysis. From the isotherm plot, shown in Figure 2A, it can be seen that both filters show a similar shape of the isotherm, featured with characteristic H1 hysteresis loops [44]. Figure 2B shows the BJH pore size distribution of the studied filters. It is seen from this graph that the filters feature different pore size distributions and pore volumes. The 11 μm prefilter shows a broader size and larger pore volume than the 22 μm filter. In particular, the pore size distribution of 11 μm filters ranges from about 2 nm to about 80 nm, whereas that of 22 μm virus filter ranges between 2 nm to about 50 nm with a tiny pore fraction between 50 and 80 nm. It is interesting to note that the main difference between the two filters comes from pores above 16 nm, whilst in the region between 2 and 16 nm the difference between two filters is insignificant. The pore size mode of the 11 μm filter is around 30 nm, whereas that of the 22 μm filter is around 20 nm. The observed differences between the filters were caused by differences in the drying kinetics between the filters as shown previously by Gustafsson and Mihranyan [37]. The observed differences in the pore size distribution are key for performance of the filters as it will be evident from the discussion below.

**Figure 3:**
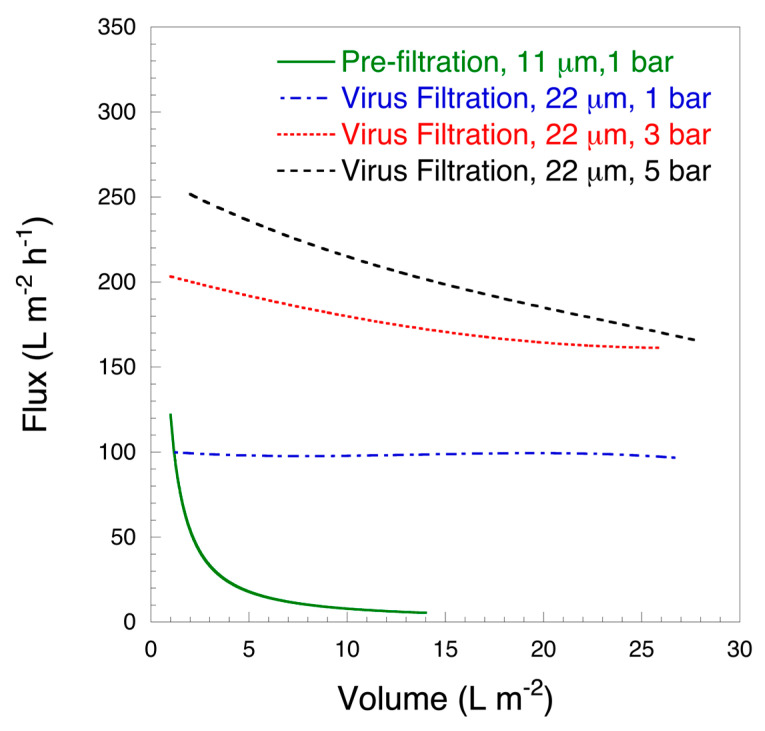
Representative flux curves for 10 mg mL^−1^ HSA solution at pH 7.4. Feed volume 14.4 L m^−2^ (prefiltration) or 28.8 L m^−2^ (filtration) (n = 3).

Figure 3 shows the flux curves of the studied filters and Table 1 summarizes the protein recovery data. It is seen in Table 1 that the overall protein recovery was above 90%. If the HSA solution was filtered without prefiltration, the filter was rapidly fouled and the flux dramatically decreased. On the other hand, if the solution was prefiltered through the 11 μm filter, the protein throughput and flux properties were substantially improved when filtered through the 22 μm filter. For prefiltration with the 11 μm filter, when the permeate volume reached 5 L m^−2^ the flow rate quickly decreased from the initial 121.6 L m^−2^ h^−1^ to about 16.7 L m^−2^ h^−1^ and then slowly decreased to 6.7 L m^−2^ h^−1^. It should be noted that protein recovery after prefiltration was 94.3 ± 1.5%, suggesting that the fraction of protein causing fouling was relatively small. It should be noted that for practical applications in order to avoid rapid fouling of the prefilter and to maintain high throughput, the sizing of the prefilter can be adjusted, as is normally done in industry, given the high cost-efficiency of the prefilter.

For filtration with the 22 μm filter, the flow rate varied depending on the applied overhead pressure. Under a constant pressure of 1 bar, the entire filtration flow rate was relatively stable at about 100 L m^−2^ h^−1^. After filtering at 28.8 L m^−2^, it only decreased to 96.7 L m^−2^ h^−1^ and the protein recovery rate was 90.7 ± 6.6% from the feed HSA content, i.e., prior to prefiltration, calculated Vmax ≥ 1000. Under a constant pressure of 3 bar, the filtration flow rate of 28.8 L m^−2^ slowly decreased from the initial 203.3 L m^−2^ h^−1^ to 161.7 L m^−2^ h^−1^ and the protein recovery rate was 92.3 ± 0.6% with a calculated Vmax 110.0 ± 2.5. At a constant pressure of 5 bar, the flow rate slowly decreased from the initial 252.5 L m^−2^ h^−1^ to 165.8 L m^−2^ h^−1^, and the protein recovery was 91.3 ± 3.5% with a calculated Vmax of 63.6 ± 5.8. The protein loss in the second filtration step was within the standard deviation range between the different groups and the main loss of about 5.6 ± 1.5% occurred during prefiltration.

**Figure 4:**
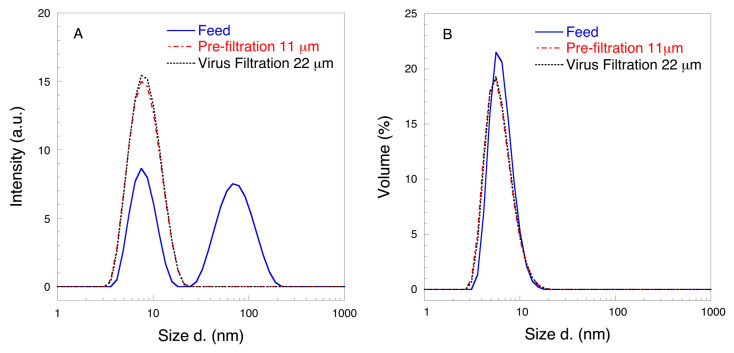
Representative dynamic light scattering (DLS) intensity (**A**) and volume (**B**) distributions of 10 mg mL^−1^ HSA at pH 7.4 (n = 3).

To understand the observed improvement in flow properties after two-step filtration, DLS and SE-HPLC analyses were performed, as discussed below. Figure 4 shows the intensity and volume distribution DLS profiles. It is seen from Figure 4A that the feed sample showed bimodal particle size distribution with peaks located at about 7.5 and 68 nm, respectively. After prefiltration with the 11 μm filter, the peak at around 68 nm could not be detected, whereas the intensity of the peak at 9 nm increased. It should be noted that the largest detected particle size was around 20 nm in the prefiltrate sample. No changes were observed after 22 μm filtration compared to 11 μm prefiltration as the distribution was monomodal. It is interesting to note that the volume distribution DLS profiles did not suggest the presence of significant number of larger particles in the feed. Nonetheless, there was a visible shift in the peak position to smaller size in the prefiltrate and permeate samples as compared to the feed sample. The latter suggests that the quantity of HSA aggregates in the feed solution was indeed low, but these aggregates, which arise during pasteurization [28,29,32], could still cause rapid filter fouling as was discussed in the Introduction section. Following filtration through the 11 μm filter and then subsequently the 22 μm filter, the peak shifted to smaller sizes. No differences were observed in the DLS distribution profiles of filtrates processed at 1, 3 and 5 bar.

**Figure 5:**
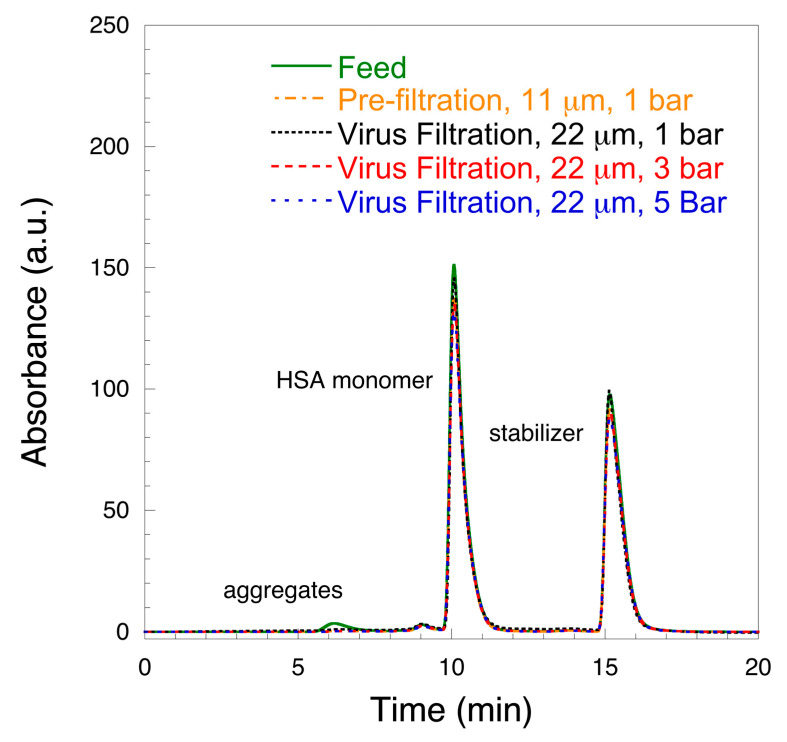
Size-exclusion high performance liquid chromatography (SE-HPLC) of 10 mg mL^−1^ HSA solution at pH 7.4.

Figure 5 shows the SE-HPLC profiles of the studied samples. As it is seen in the graph, the feed sample featured two major and two minor peaks. The major peaks were HSA monomer and stabilizer whereas the minor peaks were HSA dimer and aggregates, respectively. After prefiltration, the aggregates were removed and HSA existed mainly as a monomer. No further changes were observed after filtration through the 22 μm filter at any of the studied pressures. Furthermore, it was seen from the SE-HPLC profile that the intensity of the monomer peak did not significantly change after each respective filtration step compared to feed, which is concordant with the results of the total protein biuret assay in Table 1 and DLS volume distributions in Figure 4B. The results of SE-HPLC analysis were confirmed by ÄKTA-chromatography over Sephacryl-gel column, see Supplementary Materials, Figure S1.

**Figure 6:**
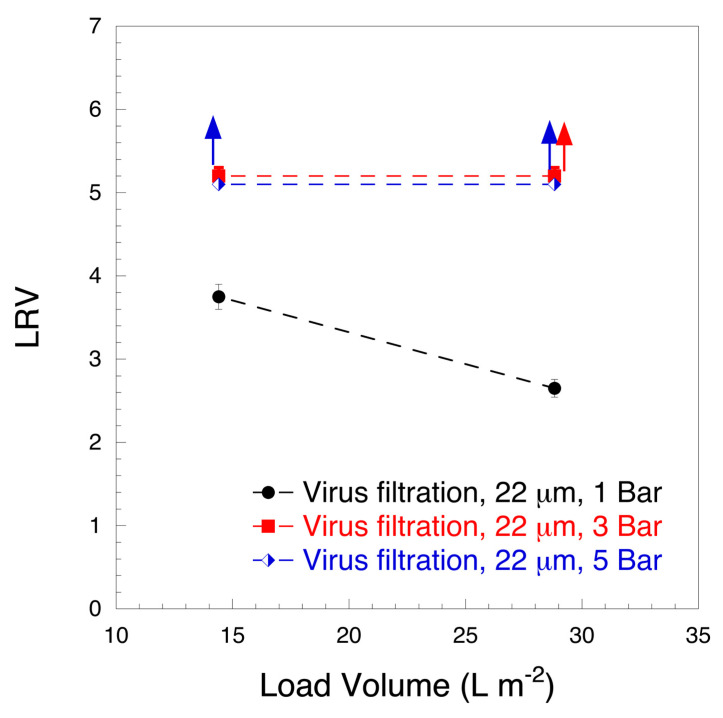
Log_10_ reduction value (LRV) vs. load volume (L m^−2^) of 10 mg mL^−1^ HSA spiked with ΦX174 (28 nm) bacteriophage at pH 7.4. (n = 3). Red and blue arrows correspond to 3 and 5 bar experiments, respectively, and indicate that no plaque forming units were detectable in the agar plate for the permeate fraction.

To verify the virus clearance of the 22 μm filter, HSA prefiltered solution was spiked with ΦX174 phage. Figure 6 shows the results of the virus clearance tests. At 1 bar constant pressure, an LRV ≤ 3.75 was observed. As the load volume increased from 14.4 L m^−2^ to 28.8 L m^−2^, virus clearance decreased from 3.75 LRV to 2.65 LRV when operated at 1 bar. Furthermore, filtration at 1 bar was more time consuming than that at 3 or 5 bar. At 3 bar and 5 bar, constant pressure virus clearance was >5 LRV and stable for increasing load volume fractions. The results of Figure 6 suggest that a higher constant pressure during virus filtration is associated with better virus clearance and a faster flow rate with little effect on protein recovery. Better virus clearance at an overhead pressure ≥3 bar is concordant with previously published data [34]. The observed improved clearance at higher pressure is related to a combined effect of compaction of the filter at higher pressure [45] and decreased tendency to Brownian motion [46].

## 4. Conclusions

The removal of HSA aggregates dramatically improved the flow properties of the filter, enabling high protein throughput and virus clearance. A distribution of pore sizes between 40 nm and 60 nm, present in the 11 μm prefilter and absent in the 22 μm virus filter, played a crucial part in removing HSA aggregates. With respect to virus filtration, 1 bar constant pressure filtration showed poor removal ability of ΦX174 bacteriophage (28 nm), i.e., LRV ≤ 3.75, while constant pressure filtration at 3 bar and 5 bar achieved LRV > 5 and overall fast filtration. It is possible to more safely and quickly eliminate virus risk of plasma products while maintaining the biological activity of its various components.

## Figures and Tables

**Table 1:** Total protein biuret assay and Vmax calculated with intermediate fouling model (n = 3).

Sample	Pressure, Bar	HSA Recovery, %	Vmax, L m^−2^
Prefiltration, 11 μm	1	94.3 ± 1.5	/
Virus filtration, 22 μm	1	90.7 ± 6.6	≥1000
Virus filtration, 22 μm	3	92.3 ± 0.6	110.0 ± 2.5
Virus filtration, 22 μm	5	91.3 ± 3.5	63.6 ± 5.8

## Supplementary Materials

The following are available online at https://www.mdpi.com/2227-9059/8/7/209/s1, Figure S1: SEC-ÄKTA chromatography of 10 mg mL^−1^ HSA solution at pH 7.4. SEC-ÄKTA chromatography. Protein Purification of HSA-PBS solution using Size Exclusion Chromatography (ÄKTA START) instrument. Selected chromatographic column (Mw 40–20,000 kDa; HiPrep 26/60 Sephacryl S-500HR, GE, Uppsala, Sweden) was used with a flow velocity of 1 mL min^−1^. The column was equilibrated with 0.5 cV of PBS buffer and then 3.2 mL 1 wt. % HSA-PBS solution was passed through the column using 3 cV. The purified solution was collected using peak fractionating in 10 mL falcon tubes, fractions were collected when absorbance was ≥5 mAU.

## Abbreviations

BSA	bovine serum albumin
CHO	Chinese hamster ovary cells
CPV	canine parvovirus
DLS	dynamic light scattering
DMEM	Dulbecco’s modified Eagle’s medium
HSA	human serum albumin
ITS	insulin-transferrin-selenium
IVIG	intravenous immunoglobulin
LBM	Luria-Bertani medium
LRV	log10 reduction value
MVM	minute virus of mice
PFU	plaque forming units
PBS	phosphate buffer saline
SE-HPLC	size-exclusion high performance liquid chromatography
SEM	scanning electron microscopy
xMuLV	xenotropic murine leukemia virus
