# Supplementary material for: Aggregate Removal Nanofiltration of Human Serum Albumin Solution Using Nanocellulose-Based Filter Paper
Source: Biomedicines. 2020 Jul 13;8(7):209. doi: 10.3390/biomedicines8070209 (PMC7400174; doi:10.3390/biomedicines8070209)
Supplement: Supplementary file 1 [file biomedicines-08-00209-s001.pdf]

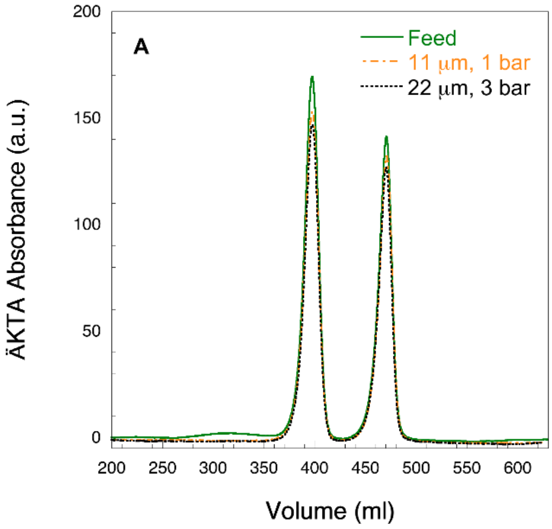

Figure S1. SEC-ÄKTA chromatography of 10 mg mL^{-1} HSA solution at pH 7.4.

Figure S1 shows a broad peak (compared with SE-HPLC curve) representing HSA aggregates in the feed curve since Sephacryl gel column has a wider pore size distribution than analytical bioZen SEC-3 column for SE-HPLC.
